# Using the 2026 Surviving Sepsis Campaign Guidelines in Practice

**DOI:** 10.62675/2965-2774.20260036

**Published:** 2026-04-10

**Authors:** Luciano Cesar Pontes Azevedo, Sheila Nainan Myatra, Naomi Hammond

**Affiliations:** 1 Hospital Israelita Albert Einstein Academic Research Organization São Paulo SP Brazil Academic Research Organization, Hospital Israelita Albert Einstein - São Paulo (SP), Brazil.; 2 Universidad Autónoma de Chile Faculty of Health Sciences Santiago Chile Faculty of Health Sciences, Universidad Autónoma de Chile, Santiago, Chile.; 3 Homi Bhabha National Institute Tata Memorial Hospital Department of Anesthesiology, Critical Care and Pain Mumbai India Department of Anesthesiology, Critical Care and Pain, Tata Memorial Hospital, Homi Bhabha National Institute - Mumbai, India.; 4 UNSW Sydney Faculty of Medicine Institute for Global Health Kensington New South Wales Australia Critical Care Division, The George Institute for Global Health, Faculty of Medicine, UNSW Sydney, Kensington, New South Wales, Australia.

Sepsis remains a leading cause of preventable mortality worldwide, with outcomes mostly influenced by early recognition, timely antimicrobials, appropriate resuscitation, and supportive care.^(1)^ The Surviving Sepsis Campaign (SSC) guidelines were launched in the early 2000s in response to the unacceptably mortality associated with sepsis worldwide.^(2)^ After successive iterations of clinical evidence and expert consensus, the guidelines introduced care bundles and time-sensitive interventions that improved sepsis management. The most recent SSC 2026 guidelines provide evidence-based recommendations that emphasize protocolized early care while acknowledging uncertainty in domains such as fluids, vasopressor timing, adjunctive therapies, and the adaptation of recommendations to local constraints.^(3)^ This manuscript reviews the key new recommendations on SSC 2026, its controversies, the impact in resource-constrained settings, and implications for future care.

## NEW RECOMMENDATIONS

There are major updates in the 2026 SSC guideline (Table 1), which emphasize a patient-centered approach to the early identification and management of sepsis. Immediate screening with standardized tools in the prehospital setting is encouraged, while collecting blood cultures before antimicrobial administration underscores the importance of pathogen identification. Initial hemodynamic stabilization prioritizes crystalloid resuscitation with immediate vasopressor infusion for persistent hypotension. While the initial mean arterial pressure threshold of 65mmHg was maintained for general patients, older adults may benefit from lower arterial pressure targets. Antimicrobial strategies highlight early use in patients with delayed access to in-hospital care, judicious empirical coverage specific to multidrug-resistant or anaerobic risk profiles, and selective use of rapid diagnostics guided by local epidemiology and stewardship principles. A controversial recommendation for the use of selective digestive decontamination in scenarios of low antimicrobial resistance was also issued, as this intervention may be associated with ecological harm and the selection of resistant organisms.^(4)^ Hemodynamic monitoring recommendations reflect current uncertainty, allowing use of either invasive or noninvasive arterial pressure modalities and underscoring the lack of definitive evidence for advanced cardiac output monitoring or adjunctive agents such as methylene blue or midodrine. In respiratory support, the guidance favors high-flow nasal cannula as the initial device for sepsis-associated respiratory failure, alongside individualized oxygen targets and consideration of awake proning. The guidelines also suggested using higher tidal volumes (6 - 8mL/kg) in the absence of lung injury. The adjunctive therapies section demonstrates the lack of new treatments, since there are new recommendations against routine antipyretics for improving clinical outcomes (while these drugs may be used for symptom relief) and against probiotics and beta blockers for treatment of sepsis and septic shock. Finally, emphasis is placed on transitions of care, including advance care planning, pharmacist-led medication reconciliation, structured post-discharge support, and access to mental health services for survivors.

**Table 1:** Key new recommendations of the Surviving Sepsis Campaign guidelines 2026

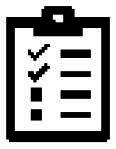	**Screening and early management**
Use a sepsis screening tool for critically ill adults *en route* to the hospital by ambulance or flight	
Collect blood cultures as soon as possible, ideally before the administration of antimicrobial therapy	
In sepsis-induced hypotension, use an initial intravenous crystalloid fluid bolus resuscitation followed by early vasopressor support if hypotension persists	
In patients with septic shock aged 65 years or older, aim for an initial MAP range of 60 - 65mmHg	
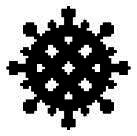	**Infection**
In probable sepsis with hypotension and a longer time to in-hospital medical evaluation (over 60 minutes), administer antimicrobial therapy in an ambulance or flight	
In sepsis at high risk of infection with a specific multidrug resistant (MDR) pathogen, use empirical antimicrobial therapy with MDR coverage	
In sepsis at high risk for anaerobic infection, use empiric antibiotic with anaerobic coverage	
Use pathogen-specific rapid diagnostic tests in selected patients based on clinical features and local scenario	
In mechanically ventilated sepsis in units with low antimicrobial resistance, use selective decontamination of the digestive tract	
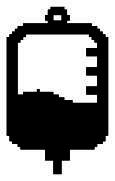	**Hemodynamic management**
In septic shock, use either invasive or non-invasive blood pressure monitoring	
In sepsis who have already received fluid resuscitation with 30mL/kg and have persistent hypoperfusion, use either a liberal or a restrictive fluid strategy	
In septic shock, there is insufficient evidence to make a recommendation on using minimally invasive or non-invasive cardiac output monitoring	
In septic shock and concomitant cardiac dysfunction, use either norepinephrine or epinephrine as the first vasopressor	
In refractory septic shock and escalating vasopressor requirements, there is insufficient evidence to make a recommendation on IV methylene blue	
In septic shock and ongoing requirement for vasopressors, there is insufficient evidence to make a recommendation on the use of oral midodrine	
In septic shock, do not use beta-blockers	
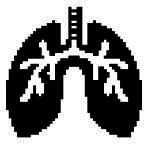	**Respiratory support**
In sepsis, measure oxygenation by either pulse oximeter (SpO_2_) or arterial blood gas (SaO_2_) in conjunction with physical examination	
In sepsis and acute hypoxemic respiratory failure, titrate FiO_2_ to target either higher or lower oxygen levels depending on patient and resource factors	
In sepsis and acute hypoxemic respiratory failure, use high flow nasal cannula therapy over conventional oxygen and non-invasive ventilation	
In sepsis and acute hypoxemic respiratory failure patients who are not intubated, use a trial of awake proning	
In sepsis and acute hypoxemic respiratory failure without ARDS, use a tidal volume of 6 - 8mL/kg ideal body weight	
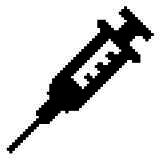	**Additional and adjunctive therapies**
In patients with sepsis and fever, do not use antipyretic therapy, either pharmacologic or surface cooling, for improving clinical outcomes	
In sepsis, do not use probiotics	
In septic shock after the acute resuscitation phase, use active fluid removal	
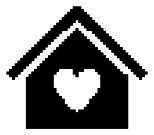	**Goals and transitions of care**
Implement strategies to ensure that patients being discharged from the hospital after sepsis can execute advanced directives	
In sepsis, perform comprehensive medication reconciliation using a pharmacist-based approach at transitions in care	
Implement strategies to support sepsis survivors and their families during the post-hospital recovery	
In sepsis survivors, offer services that support mental health after hospital discharge	

## CONTROVERSIES

Despite their preeminent role in standardizing sepsis care worldwide, the SSC guidelines are not free of controversy. One major area of debate concerns the strength of recommendations derived from low- or very-low-certainty evidence. The guidelines rely mostly on recommendations with low-quality evidence, and only a minority are supported by randomized controlled trials.^(5,6)^ The societies justify their recommendations by prioritizing early intervention and standardized care to improve outcomes, but acknowledge that many recommendations are based on expert consensus rather than robust clinical trial data. Critics argue that treatment based mostly on expert consensus and guidelines promotes protocolized care that may not be appropriate for all patients, potentially discouraging individualized clinical judgment.

Fluid resuscitation targets represent another issue, particularly the use of a fixed volume of intravenous crystalloids. Even though this recommendation has been downgraded in recent iterations, this approach may still fail to account for patient heterogeneity and may contribute to fluid overload and worse outcomes.^(7)^ Nevertheless, the suggestion to use dynamic measures, including response to a passive leg raise or a fluid bolus using stroke volume, stroke volume variation, pulse pressure, or pulse pressure variation to guide fluid resuscitation over physical examination or static measures alone, is reassuring.

Other major criticisms concern the previous prioritization of short-term mortality outcomes over patient-lefted measures, such as long-term outcomes and quality of life. This last issue has been minimized in the last two iterations, which now comprise specific sections for goals of care and long-term outcomes.^(2,3)^ These controversies reflect the imbalance between protocolization and personalization in sepsis care, underscoring the need for future guidelines to balance evidence with contextual adaptation.

## USING THE GUIDELINES IN RESOURCE-LIMITED SETTINGS

A major criticism of previous iterations of the SSC guidelines was the unfeasibility of several recommendations in resource-limited settings (RLS). Issues precluding the broad adoption of the recommendations include inadequate resources, differences in disease etiology, and differences in case mix across sepsis populations. Additionally, international guidelines do not address the safe management of critically ill patients with sepsis in the absence of intensive care unit beds, when factors such as the minimum monitoring, triggers for escalation of care, and safe transport are important.^(8,9)^ A recent international expert consensus using Delphi methodology developed expert clinical practice statements providing guidance on the management of sepsis in RLS, complementing the SSC guidelines where barriers to the effective implementation of current international guidelines exist or where guidance for specific sepsis management issues relevant in these settings is lacking.^(8)^

For the 2026 version of the SSC guidelines, the geographic diversity of the panelists was improved, with 23 countries represented, and 38% of them are currently or previously practicing in a low- or middle-income country.^(3)^ The applicability of recommendations in RLS was discussed in accordance with recent GRADE orientations. Most recommendations now include comments on their relevance to RLS, which may help healthcare workers adapt them to their local care.

Implementing sepsis guidelines in RLS requires prioritization, contextual adaptation, and pragmatic use of available resources. Elements with a major impact, such as early recognition using bedside criteria and prompt antibiotic administration, should be emphasized over technology-based management.^(10-13)^ Protocols must be simplified and integrated into routine workflows. Training frontline staff and using checklists can also improve adherence and consistency of care.

## IMPLICATIONS FOR THE FUTURE

The future of sepsis treatment and clinical guidelines is moving from a protocolized approach toward more individualized care. While early recognition, prompt antimicrobials, fluid resuscitation, and organ support remain the treatment cornerstones, growing evidence indicates that sepsis is a heterogeneous syndrome rather than a single disease entity.^(10)^ As a result, future guidelines must emphasize biological phenotyping and endotyping, allowing therapies to be tailored to host immune responses and disease trajectories instead of the "one size fits all" approach.

In this regard, advances in diagnostics will probably play a major role. Rapid molecular tests, host-response biomarkers, and point-of-care technologies are likely to shorten time to diagnosis, improve pathogen identification, and support more rational antimicrobial use.

Another key direction is the integration of Artificial Intelligence and clinical decision support systems. Predictive models based on real-time physiological data may assist clinicians in early detection and risk stratification. Pending prospective validation, such tools may become incorporated into guideline recommendations as decision aids rather than replacements for clinical judgment.

Implementation science studies will also shape future guidelines, since even the best guidelines have limited impact without effective adoption across diverse healthcare settings. A major challenge is implementing these new strategies in RLS, as their structural difficulties in diagnostics and supportive care may hinder the application of sepsis’ precision treatment. Moreover, emphasis on research and recommendations that reflect global resource availability is paramount. Together, these trends will improve a patient-lefted and equitable approach to sepsis care.

## Data Availability

The contents are already available.
